# Polymer Composite Hydrogel Based on Polyvinyl Alcohol/Polyacrylamide/Polybenzoxazine Carbon for Use in Flexible Supercapacitors

**DOI:** 10.3390/polym16111463

**Published:** 2024-05-22

**Authors:** Thirukumaran Periyasamy, Shakila Parveen Asrafali, Mobinul Islam, Gazi A. K. M. Rafiqul Bari, Jaewoong Lee

**Affiliations:** 1 Department of Fiber System Engineering, Yeungnam University, 280 Daehak-Ro, Gyeongsan 38541, Republic of Korea; thirukumaran@ynu.ac.kr (T.P.); shakilaparveen@yu.ac.kr (S.P.A.); 2 Department of Energy & Materials Engineering, Dongguk University, Seoul 04620, Republic of Korea; mobin85@dongguk.edu; 3 School of Mechanical Smart and Industrial Engineering, Gachon University, 1342 Seongnam-daero, Sujeong-gu, Seongnam-si 13120, Republic of Korea

**Keywords:** composite polymer hydrogels, polybenzoxazine-based carbon, hetero-atom conductive gels, flexible SCs

## Abstract

Polymer gels are cross-linked polymer networks swollen by a solvent. These cross-linked networks are interconnected to produce a three-dimensional molecular framework. It is this cross-linked network that provides solidity to the gel and helps to hold the solvent in place. The present work deals with the fabrication of polybenzoxazine carbon (PBzC)-based gels that could function as a solid electrode in flexible supercapacitors (SCs). With the advantage of molecular design flexibility, polybenzoxazine-based carbon containing different hetero-atoms was synthesized. A preliminary analysis of PBzC including XRD, Raman, XPS, and SEM confirmed the presence of hetero-atoms with varying pore structures. These PBz-carbons, upon reaction with polyvinyl alcohol (PVA) and acrylamide (AAm), produced a composite polymer hydrogel, PVA/poly (AAm)/PBzC. The performance of the synthesized hydrogel was analyzed using a three-electrode system. PVA/poly (AAm)/PBzC represented the working electrode. The inclusion of PBzC within the PVA/poly (AAm) matrix was evaluated by cyclic voltammetry and galvanostatic charge/discharge measurements. A substantial increase in the CV area and a longer charge/discharge time signified the importance of PBzC inclusion. The PVA/poly (AAm)/PBzC electrode exhibited larger specific capacitance (C_s_) of 210 F g^−1^ at a current density of 0.5 A g^−1^ when compared with the PVA/poly (AAm) electrode [Cs = 92 F g^−1^]. These improvements suggest that the synthesized composite hydrogel can be used in flexible supercapacitors requiring light weight and wearability.

## 1. Introduction

Portable electronics are everywhere today, and along with them comes the constant need for safe, flexible, and efficient ways to store energy. Supercapacitors are considered a promising solution for safe and efficient energy storage, as they can hold more power and recharge quickly for many cycles [1,2,3]. There are two main types of supercapacitors: electrochemical double-layer capacitors (EDLCs) and pseudo-capacitors. EDLCs store energy in the form of the physical separation of charges on their electrodes [4,5,6]. Their working capability depends highly on the materials used for the electrodes. So far, researchers have focused on carbon materials including fullerenes, nanotubes, and activated carbons to be used as EDLC electrodes. However, these materials fail to store enough energy and to release the energy fast enough for widespread use. Finding new carbon materials with better performance is currently a major hurdle [7,8,9,10,11]. As the search for better and new materials is not limited only to carbon-based materials, scientists worldwide have also used metal oxides, conducting polymers, and composite materials in addition to carbon materials [12]. These materials have unique properties that might outperform carbon and unlock new possibilities for supercapacitors. Apart from finding the new materials, the design strategy of electrodes also plays a crucial role [13,14,15]. Therefore, research is focusing on different techniques, like nano structuring and surface functionalization, to combine different materials, so that fabricated devices can store more energy while maintaining their stability and reliability. This will ultimately lead to the next generation of supercapacitors that can meet the ever-increasing demands of our electronic devices [16,17].

Hydrogels, renowned for their biocompatibility and water-retaining properties, have become indispensable in wound care, drug delivery systems, and skincare products like face masks. However, their utility has been limited due to their relatively low mechanical strength, restricting their application in fields requiring durability. Fortunately, recent decades have witnessed significant progress in the development of high-strength hydrogels. Various strategies have been explored to bolster their mechanical properties, including the incorporation of covalent or ionic cross-linking, nanoparticles, and the construction of double networks (DNs). Yet, the manufacturing of these advanced hydrogels often presents challenges, impeding their widespread adoption. Additionally, they tend to be soft and lack a high initial stiffness, or elastic modulus. Presently, the construction of double networks stands out as the most effective method for achieving substantial strength [18,19,20]. Double networks involve the combination of two polymers that interact through chemical bonds, hydrophobic interactions, hydrogen bonding, or microcrystalline networks. These interactions contribute to superior mechanical strength compared to other methods, with initial moduli ranging from 0.2 to 1.0 MPa and compressive strengths of 20 to 60 MPa. However, there is a trade-off to consider. The strong interactions within double networks limit their free volume, thereby reducing their ability to absorb water. For instance, Yang et al. [21] developed a double-network hydrogel with impressive strength (initial modulus of 1.2 MPa, compressive strength of 60 MPa), but its low swelling ratio (around 100%) restricts its water absorption capabilities. In essence, scientists are striving to strike a balance between high-strength properties and excellent water absorption in hydrogels. This equilibrium is vital for expanding their applications across diverse fields [22].

The electrochemical performance of the flexible supercapacitor depends on both the electrode and electrolyte parts. And so, in recent years, gel electrolytes based on poly(vinyl alcohol) (PVA), such as PVA/LiCl, PVA/KOH, and PVA/H2SO4, have been widely used. More recently, to increase their conductivity and stretchability, polyacrylic acid (PAA)- and polyacrylamide (PAM)-based gel electrolytes have been synthesized. Even though these electrolytes could solve the problem of stretchability, they still possess low conductivity. In the case of gel electrolytes based on ionic liquids, increased conductivity was observed but with low stretchability. Therefore, gel electrolytes with high conductivity and appreciable stretchability will be suitable for flexible SCs [2,3,4,5,6,7].

Expanding on previous investigations, we have pioneered the development of an innovative polymer gel electrolyte. This electrolyte amalgamates polyvinyl alcohol (PVA), acrylamide, and a novel benzoxazine monomer (Bzo) containing curcumin and stearylamine. Polybenzoxazines (PBzs) are an advanced form of phenol–formaldehyde resins, and has several advantages, including minimal water absorption, minimal shrinkage during formation, not requiring catalysts for polymerization, and maintaining its shape effectively. Moreover, its molecular structure can be tailored to suit specific applications. The synthesis process was carried out through a Mannich condensation reaction, resulting in the formation of a benzoxazine monomer capable of self-polymerization upon heating, forming a polybenzoxazine (PBz) structure rich in hydroxyl (–OH) and amine (–NH_2_) groups. Upon carbonization, a polybenzoxazine based carbon (PBzC) is produced, which then integrates seamlessly with PVA and acrylamide, giving rise to a robust PVA/polyacrylamide/PBzC hydrogel. The stability of this hydrogel is underpinned by strong hydrogen-bonding interactions between the –OH groups of PVA, the –C=O and –NH_2_ groups of polyacrylamide, and the hetero-atoms of PBzC. Additionally, the presence of hetero-atoms in PBzC and polyacrylamide enhances their affinity for PVA, further reinforcing the hydrogel’s structural integrity. The performance of this composite hydrogel was investigated in a three-electrode system. Through detailed electrochemical analysis, the performance of this composite hydrogel, as well as its energy-storage capabilities and stability under various conditions, have been characterized and discussed extensively.

## 2. Materials and Methods

### 2.1. Materials

Curcumin, stearylamine, and paraformaldehyde were procured from Sigma-Aldrich (St. Louis, MO, USA). Dimethyl sulfoxide (DMSO), N,N-dimethylacetamide (DMAc), and sodium hydroxide (NaOH) were provided by Duksan Chemicals Co., Ltd., Ansan-si, Republic of Korea. Polyvinyl alcohol (PVA) with a molecular weight (M_w_) range of 146,000−186,000 and ammonium persulfate (APS, 99%) were sourced from Daejung Chemical Company, Ltd., Seoul, Republic of Korea. Acrylamide (AAm), N,N′-methylenebisacrylamide (MBA, 99%), and hydrochloric acid (HCl, 35%) were purchased from Alfa Aesar. All chemicals were utilized without further purification.

### 2.2. Synthesis of Polybenzoxazine-Based Carbon (PBzC)

**Scheme 1:**
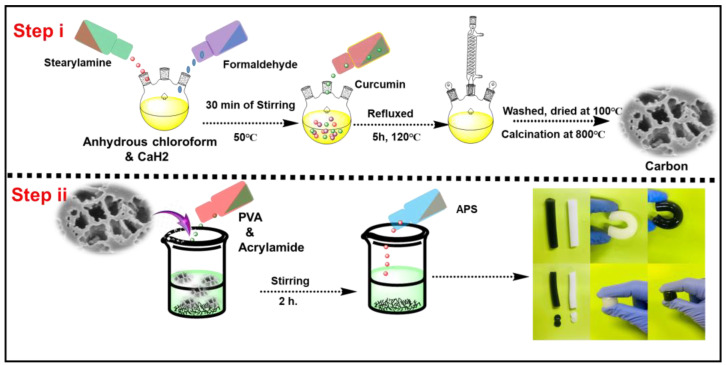
Synthesis of PBzC and PVA/poly (AAm)/PBzC hydrogel.

The synthesis of curcumin-stearylamine-based benzoxazine (C-st) can be achieved in a single reaction process. To begin, a round-bottomed flask was charged with anhydrous chloroform, curcumin, paraformaldehyde, stearylamine, and calcium hydride. After ensuring thorough mixing of the reactants, the flask was heated to reflux for eight hours while continuously stirring the reaction mixture. Once the reaction was complete, the mixture was allowed to cool down to room temperature. Subsequently, filtration separated the liquid phase, and the filtrate was concentrated under reduced pressure to eliminate excess chloroform. The remaining residue was then treated with an excess of methanol, inducing precipitation of the desired C-st product. The collected precipitate was thoroughly washed with deionized water to remove any impurities and finally dried under vacuum at 60 °C for a day to obtain pure C-st with a high yield of 85% (Scheme 1). The obtained benzoxazine monomer was heated in a stepwise manner in an oven at 100, 150, 180, 220, and 250 °C for 4 h. Then, the cured polybenzoxazine was further carbonized under an argon atmosphere by heating at 600 °C for 5 h with a ramp rate of 1 °C min^−1^. Then, the obtained carbonized material was soaked in an aqueous KOH solution (twice the weight ratio of the carbonized material) overnight and finally filtered and dried at 120 °C. The activation process was carried out at 800 °C for 1 h in a tube furnace under flowing argon with a ramp rate of 3 °C min^−1^. The products were repeatedly washed with 1 M HCl and deionized water until the pH value of the filtrate reached about 7 and dried at 110 °C for 12 h. The obtained sample was named PBzC.

### 2.3. PVA/Poly (AAm)/PBzC Hydrogel Preparation

The hydrogel, consisting of poly(acrylamide) (AAm), poly(vinyl alcohol) (PVA), and PBzC, underwent a meticulous three-step fabrication process. Initially, AAm and PVA were dissolved in water at a precise concentration of 20 wt%, with the addition of 2 wt% of PBzC. Following this, a cross-linker (MBA, 0.1 mol%) and an initiator (APS, 0.3 mol%) were carefully introduced into the solution. The reaction was carried out at a lower temperature of 2 °C to initiate the radical polymerization of AAm. The solution was then transferred into a polypropylene tube and heated to 40 °C for 6 h, allowing it to shape into cylindrical gels. To ensure purity, the resulting gels were rinsed three times with distilled water, resulting in an impressive gel fraction of up to 95%. In the second stage, the cylindrical gels were immersed in a hydrochloric acid (HCl) solution with a pH of approximately 2 for 1 h to induce PVA crystallization. Subsequently, the gels were dried overnight at 50 °C. Finally, in the third stage, amide groups were hydrolyzed using a 5% sodium hydroxide (NaOH) solution at 65 °C for 6 h. Following this hydrolysis process, the gel was thoroughly rinsed with water to remove any remaining impurities. The hydrogel was then dried overnight at 50 °C, completing the meticulous three-stage fabrication process (Scheme 1). Following a similar procedure, another hydrogel was prepared without PBzC. The synthesized hydrogels were denoted as PVA/poly(AAm) and PVA/poly(AAm)/PBzC.

### 2.4. Instrumentation

The physicochemical properties of the benzoxazine monomer, PBzC, and the polymer gels (PVA/poly(AAm) and PVA/poly(AAm)/PBzC) were characterized accordingly. Fourier transform infrared (FT-IR) spectroscopy, conducted using a Perkin Elmer MB3000 (Hopkinton, MA, USA) instrument, was utilized to identify functional groups within the 400–4000 cm^−1^ range with a resolution of 4 cm^−1^. Nuclear magnetic resonance (NMR) spectroscopy, performed on an Agilent NMR (AVANCE NEO600 Oxford, UK, 600), provided detailed structural information about both the protons (at 600 MHz) and carbon atoms (at 150 MHz) within the samples. Thermal behavior was investigated through differential scanning calorimetry (DSC) using a TA Instruments (New Castle, DE, USA) Q200 model. Samples (5−10 mg) were heated under a nitrogen atmosphere at a rate of 10 °C/min. The mechanical properties of the hydrogels were assessed via compression tests using an Instron model 3345 tester at a rate of 10 mm/min. To prevent slippage, samples were securely affixed with glue onto sandpaper during testing. Hysteresis behavior was explored through a stress test using an Instron E300LT fatigue tester, with the same sample dimensions as the compression test, repeated 30 times. Microscopic morphology and elemental composition were examined using field emission scanning electron microscopy (FESEM) with energy-dispersive X-ray spectroscopy (EDS) on a Hitachi S-4800 (Ibaraki, Japan) at an accelerating voltage of 4 kV. Additionally, high-resolution transmission electron microscopy (HRTEM) images were obtained using a FEI-Tecnai TF-20 instrument with an accelerating voltage of 120 kV. The crystalline structure was determined by X-ray diffraction (XRD) using a PANalytical X’Pert3 MRD diffractometer with Cu Kα radiation (λ = 1.54 Å) at 40 kV and 30 mA, scanning from 10 to 80° (2θ). Raman spectroscopy (Horiba XploRA Micro-Raman) (HORIBA, Palaiseau, France SAS) was employed to identify vibrational modes within the material between 500 and 4000 cm^−1^. The specific surface area, pore size distribution, and pore volume were determined by observing nitrogen adsorption–desorption isotherms measured at −197 °C using a Micromeritics ASAP 2000. Prior to analysis, samples were degassed at 120 °C and evacuated for 8 h in flowing argon (60 sccm) at 140 °C. The Brunauer–Emmett–Teller (BET) and Barrett–Joyner–Halenda (BJH) methods were utilized to interpret the isotherm data. Finally, X-ray photoelectron spectroscopy (XPS) analysis, conducted using a Thermo Fisher Scientific K-Alpha instrument (Waltham, MA, USA), provided information about the surface elemental composition and chemical states. CasaXPS software was employed to deconvolute the high-resolution XPS spectra. All analyses were carried out at the core research support center for natural products and medical materials of Yeungnam University.

### 2.5. Electrochemical Measurements

To characterize the electrochemical properties of the polymer gels, a three-electrode system was employed. This setup utilized an Ag/AgCl reference electrode for stable potential reference and a platinum counter electrode to complete the circuit. A 1 M H_2_SO_4_ aqueous solution served as the electrolyte for ionic conductivity. Working electrodes were fabricated by directly cutting the hydrogel material into a disc shape and pressing it onto a small carbon cloth substrate (~3 mg mass). To evaluate the electrochemical performance, cyclic voltammetry (CV) and galvanostatic charge/discharge (GCD) tests were conducted. These tests were carried out within a potential window of 0 to 1.0 V, providing information on electron transfer processes during charging and discharging. Additionally, electrochemical impedance spectroscopy (EIS) was performed at the open circuit potential. This technique, applied over a wide frequency range (10^−2^−10^5^ Hz) with a small amplitude (10 mV), determined the resistance and capacitance behavior of the electrode/electrolyte interface. Finally, the specific capacitance (F g^−1^) of the hydrogel electrodes was calculated based on the discharge portion of the GCD curves. The calculation employed the equation C_s_ = IΔt/ΔVm, where C_s_ represents specific capacitance, I denotes discharge current (A), Δt signifies discharge time (s), ΔV represents the potential window (V) during discharge, and m stands for the mass (g) of the hydrogel electrode.

## 3. Results and Discussion

### 3.1. Structural Characterization of Benzoxazine Monomer (C-st)

**Figure 1:**
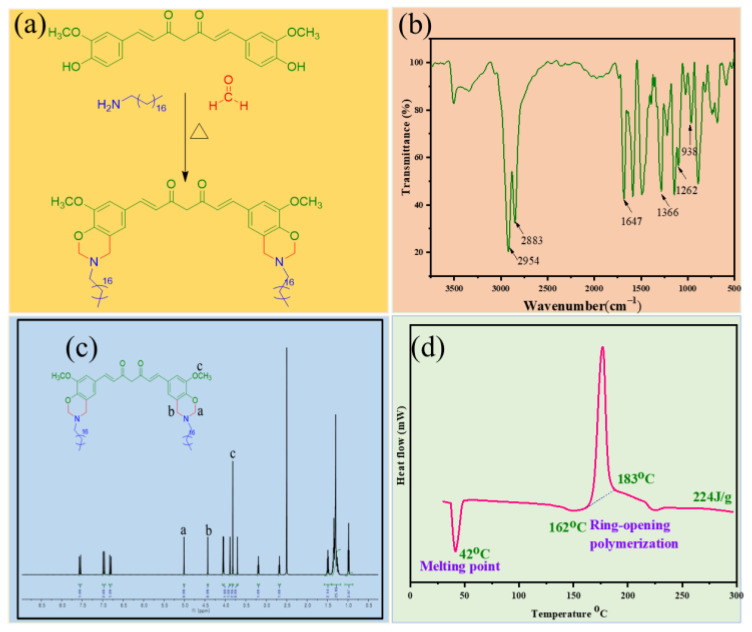
(**a**) Synthesis procedures of C-st, (**b**) FT-IR, (**c**) 1H-NMR, and (**d**) DSC of C-st.

The characterization of the synthesized C-st benzoxazine precursor (Figure 1a) confirms its successful formation through detailed analysis using both FT-IR and ^1^H-NMR spectroscopy. The FT-IR spectrum (Figure 1b) displays distinctive peaks indicating the presence of key functional groups. Specifically, the peaks at 1262 and 1254 cm^−1^ signify C-O-C stretching vibrations, characteristic of the benzoxazine ring. The peak at 938 cm^−1^ further confirms the presence of the benzoxazine ring structure. Additionally, the absorption peak at 1647 cm^−1^ indicates the amide group in chitosan. Aromatic and aliphatic characteristics are evident from peaks at 2954 and 2883 cm^−1^, attributed to the asymmetric and symmetric C-H stretching vibrations in benzene rings and stearylamine, respectively. Furthermore, the presence of a tetra-substituted benzene ring is suggested by the band at 1368 cm^−1^ [23]. The ^1^H-NMR spectrum in Figure 1c provides more detailed structural information. The signals obtained at 4.6 and 5.4 ppm correspond to protons within the oxazine ring, while the signals between 1 and 1.5 ppm come from the aliphatic chain of stearylamine. The singlet at 3.75 ppm is attributed to methoxy groups from curcumin, while doublet peaks at 2.6 and 3.2 ppm correspond to allyl protons. Finally, the multiplets between 6.3 and 7.0 ppm indicate aromatic protons from various components [24,25]. Differential scanning calorimetry (DSC) was used to study the polymerization behavior of the benzoxazine monomer. This technique measures heat flow as a function of temperature. The DSC curve of C-st in Figure 1d exhibits an endotherm, followed by an exotherm. The endotherm at 42 °C indicates the melting point of the monomer, whereas the exotherm indicates the ring-opening polymerization of the C-st monomer, with an onset of curing at 162 °C, maximum curing at 175 °C, and final curing at 183 °C. It is noteworthy that the entire curing process occurs at a lower temperature range when compared with the commercially available BA-a benzoxazine (the curing process takes place at a higher temperature around of 250 °C).

### 3.2. Structural and Morphological Analyses of PBzC

**Figure 2:**
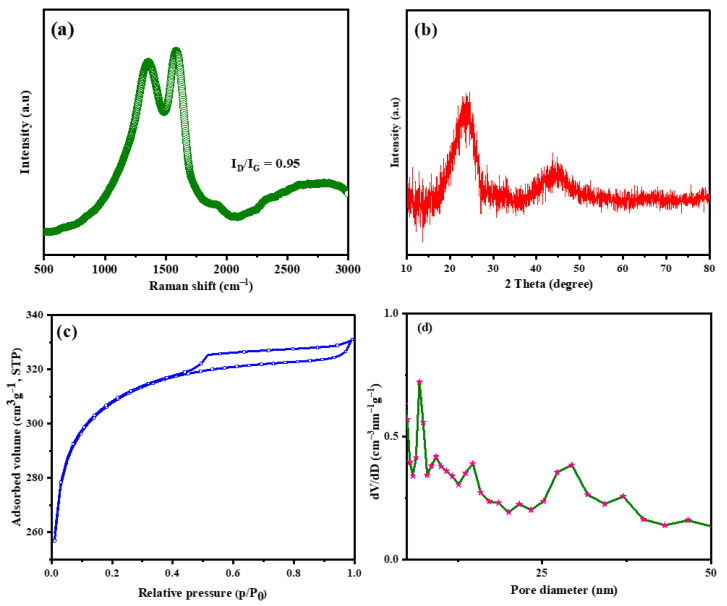
(**a**) Raman spectrum, (**b**) XRD pattern, (**c**) BET isotherm, and (**d**) pore size distribution of PBzC.

To gain insights into the structure and graphitic properties of the activated carbon materials, a combination of Raman spectroscopy and X-ray diffraction (XRD) analysis was employed. The Raman spectra depicted in Figure 2a exhibit two distinct peaks at 1354 and 1582 cm^−1^, corresponding to the ‘D’ and ‘G’ bands, respectively. These ‘D’ and ‘G’ bands represent the disordered and graphitic carbon structures, respectively. The intensity ratio (I_D_/I_G_) of these peaks was calculated to be 0.95, indicating a well-ordered structure with a significant degree of graphitization. Furthermore, the XRD patterns presented in Figure 2b reveal broad peaks at 2θ = 24.1° and 44.5°, characteristic of graphitic carbon materials, corresponding to the (002) and (100) crystal planes, respectively. By applying Bragg’s equation, the d-spacing value for the graphitic carbon was determined to be 0.37 nm. Interestingly, this value is slightly higher than that of conventional graphite, suggesting a unique structural arrangement in the activated carbon materials [26]. The combination of a well-developed graphitic structure and the observed d-spacing value indicates that these activated carbon materials possess favorable characteristics for enhanced capacitance when employed as electrodes in supercapacitors. The higher d-spacing value suggests increased interlayer spacing, which could facilitate ion transport and storage within the material, leading to improved electrochemical performance. Thus, these findings underscore the potential of these activated carbon materials for application in high-performance energy storage devices.

Figure 2c exhibits the nitrogen adsorption–desorption isotherms of the activated carbons, showcasing characteristics typical of type IV isotherms. The isotherm is distinguished by a well-defined hysteresis loop in the meso-pore range. Initially, there is a sharp ascent in N_2_ adsorption at low relative pressures (below 0.1), indicating micro-pore filling. As the relative pressure increases up to 0.9 P/P_0_, there is a gradual rise in adsorption, attributed to the filling of meso-pores with N_2_ molecules. Subsequently, a significant surge in adsorption above 0.9 P/P_0_ suggests the presence of macro-pores within the activated carbon structure [27]. Further analysis of the BET isotherm reveals a specific surface area of 987 m^2^ g^−1^ for the PBzC carbons. Interestingly, the use of KOH corrosion during the synthesis process successfully generated macro-pores within the PBzC material, with pore sizes ranging from 15 to 50 nm. Remarkably, despite the high-temperature treatments involved, the hetero-atomic functionalities like nitrogen and oxygen were retained in the PBzC material. These functionalities are often advantageous for adsorption purposes. This successful creation of macro-pores and retention of functionalities can be attributed to the inherent high chemical and thermal stability of polybenzoxazine, the precursor material used to synthesize the PBzC carbons. Additionally, the high char yield (around 50%) obtained from the polybenzoxazine (PBz) precursor allows for the efficient production of PBzC samples in good yield. This combination of factors contributes to the desirable properties of PBzC carbons for various applications.

**Figure 3:**
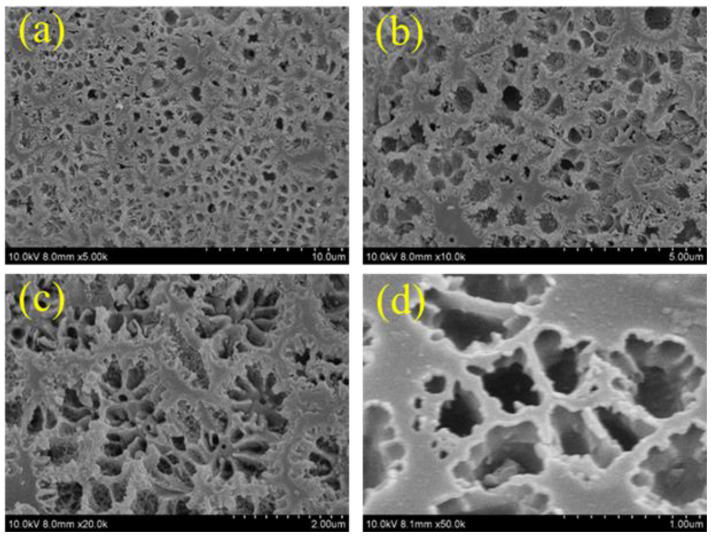
SEM images of PBzC at different magnifications at different magnifications (**a**–**d**).

**Figure 4:**
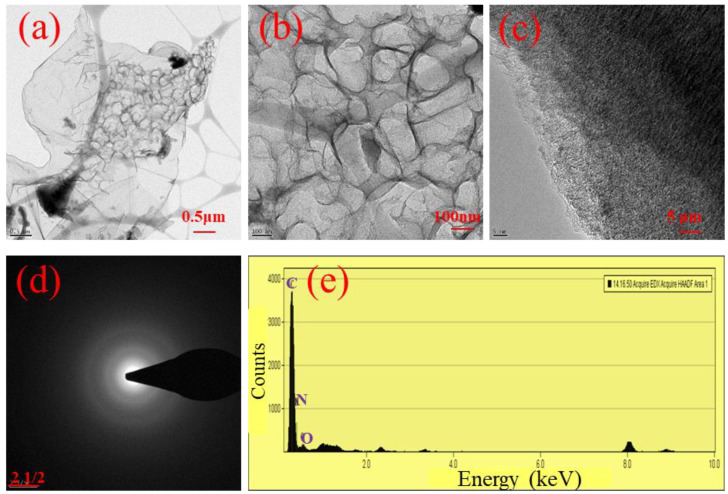
(**a**–**c**) TEM images of PBzC at different magnifications with (**d**) SAED pattern, and (**e**) EDAX.

The scanning electron microscope (SEM) images presented in Figure 3 provide insights into the surface morphology of the thermally activated PBzC. These images (Figure 3a–d) illustrate that the activation process results in a textured surface characterized by pores of diverse sizes. For instance, Figure 3a displays a porous carbon sample featuring numerous irregular micro-pores alongside a few meso-pores. This indicates the precise control of activation temperature to achieve the desired pore structure and size distribution. Further analysis using transmission electron microscopy (TEM) in Figure 4a–d confirms the morphology of the PBzC material. The TEM images reveal an interconnected open-pore network, which is highly advantageous for supercapacitor applications. This nano-architecture not only minimizes ion diffusion pathways but also facilitates continuous electron conduction. Moreover, the TEM images exhibit a more uniform arrangement of pores, suggesting the presence of sp^2^-bonded carbon, known for enhancing the electrical conductivity of materials. The hierarchical porous structures observed in the TEM images are in accordance with the findings from the SEM analysis, indicating consistent characterization of the PBzC material. This dual imaging approach underscores the controlled synthesis of PBzC with tailored porous architectures suitable for various applications, particularly in energy storage devices. The activated porous carbon materials derived from polybenzoxazine contain a relatively higher content of nitrogen and oxygen species (Figure 4e). The presence of these hetero-atoms (N and O atoms) effectively enhances the wettability and electrical conductivity of the prepared carbon materials, thereby facilitating the accessibility of electrolyte ions and enhancing their capacitance performance.

**Figure 5:**
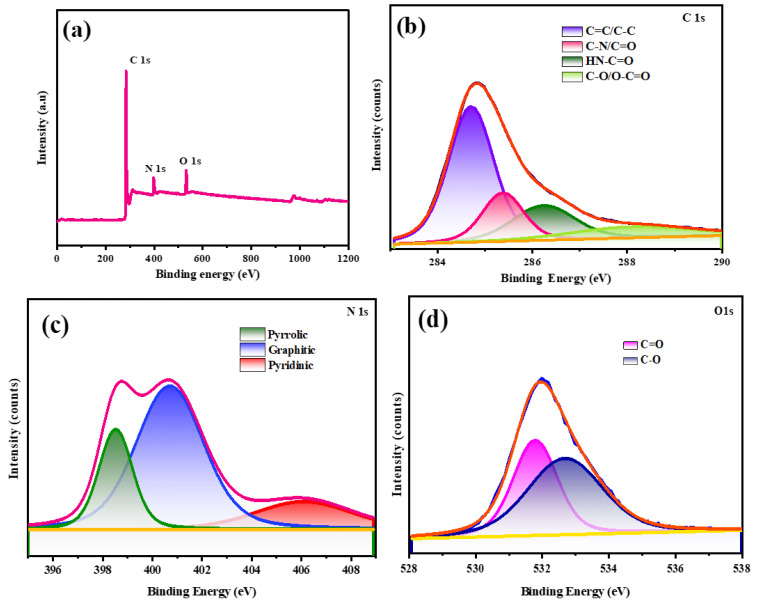
(**a**) XPS survey spectrum, and (**b**–**d**) deconvoluted spectra of PBzC.

The XPS analysis data in Figure 5 represent the chemical state of carbon, nitrogen, and oxygen species in the porous carbon materials. The XPS survey spectrum in Figure 5a exhibits three peaks corresponding to C 1s, N 1s, and O 1s at approximately 285.6, 399.1, and 531.7 eV, respectively, demonstrating the presence of these elements in the PBzC sample. This result provides strong evidence for the existence of nitrogen and oxygen atoms in the prepared carbon materials, which are expected to be derived from the benzoxazine monomer used as the carbon source. These results further indicate the absence of impurities in the synthesized nitrogen-rich porous carbon. The respective C 1s, N 1s, and O 1s signals were deconvoluted for in-depth analysis and are presented in Figure 5b–d. The fitting of the C 1s spectrum (Figure 5b) resolved into four peaks: the first peak at 284.6 eV corresponds to the hydrocarbon chains, the second peak at 285.3 eV corresponds to the carbon atoms in C–N bond, the third peak at 286.4 eV corresponds to the carbon atoms bonded with oxygen groups, and the fourth peak at 287.4 eV corresponds to the HN–C=O groups [28]. Figure 5c reveals the presence of different nitrogen species bound to the activated porous carbons. Three distinct nitrogen species were observed: at 398.3 eV due to pyrrolic nitrogen, at 400.4 eV due to graphitic nitrogen, and at 405.2 eV due to pyridinic nitrogen [29].

The O 1s spectrum (Figure 5d) deconvolutes into two binding energy peaks appearing at 531.4 and 533.0 eV, revealing the presence of quinone (Ph=O) and phenolic hydroxyl or ether (C-O-C), respectively. Among these oxygen functional groups, quinone groups in the carbon matrix are not electrochemically active in reversible redox reactions in an alkaline medium. However, phenolic hydroxyl (by reduction) and hydroxyl or ether (by deprotonation) exhibit quasi-reversible pseudo-capacitance [30]. Hence, the prepared PBzC with high contents of phenolic hydroxyl or ether oxygen and carbonyl oxygen can generate high pseudo-capacitance in electrochemical reactions.

### 3.3. Characteristic Analysis of PVA/Poly(AAm) and PVA/Poly(AAm)/PBzC Hydrogels

**Figure 6:**
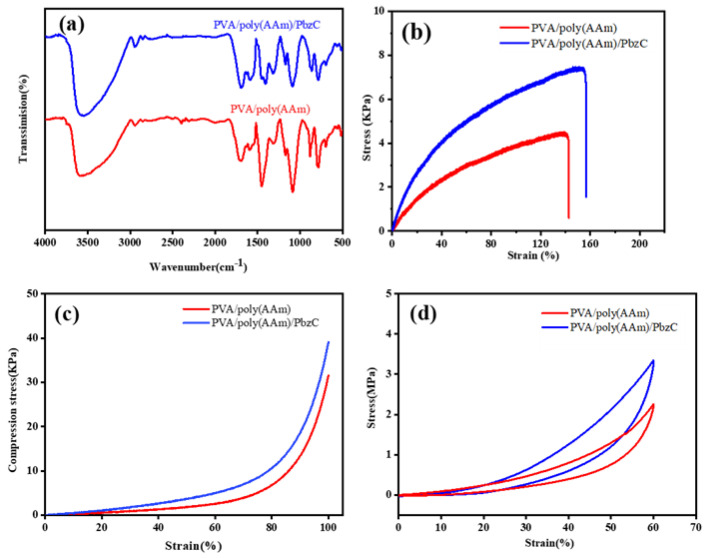
(**a**) FT-IR, and mechanical properties determined through (**b**–**d**) tensile and compression studies of the hydrogels.

The FT-IR analysis depicted in Figure 6a provides compelling evidence of the successful integration of PBzC into the hydrogel matrix. The characteristic peaks associated with amide groups (NH_2_ stretching at 3477 and 3170 cm^−1^, C=O stretching at 1690 cm^−1^, and N-H bending at 1572 cm^−1^) become weakened upon the addition of PBzC. This suggests the conversion of amides to carboxylate ions, a transformation further supported by the intensified COO- peaks at 1454 and 1172 cm^−1^. These alterations unequivocally demonstrate the chemical interaction between PBzC and the hydrogel network [31].

Figure 6b–d highlight the substantial enhancement in mechanical properties conferred by the incorporation of PBzC. The stress–strain curves reveal a remarkable increase in tensile strength, with PVA/poly (AAm)/PBzC exhibiting a strength of 7.8 KPa, compared to only 3.9 KPa for PVA/poly (AAm). Similarly, the compressive strength shows a significant improvement, with PVA/poly (AAm)/PBzC reaching 39 KPa, compared to 32 KPa for PVA/poly (AAm). These findings underscore the reinforcing effect of PBzC on the hydrogel structure, positioning it as a promising candidate for applications that demand superior mechanical properties.

### 3.4. Electrochemical Measurements

**Figure 7:**
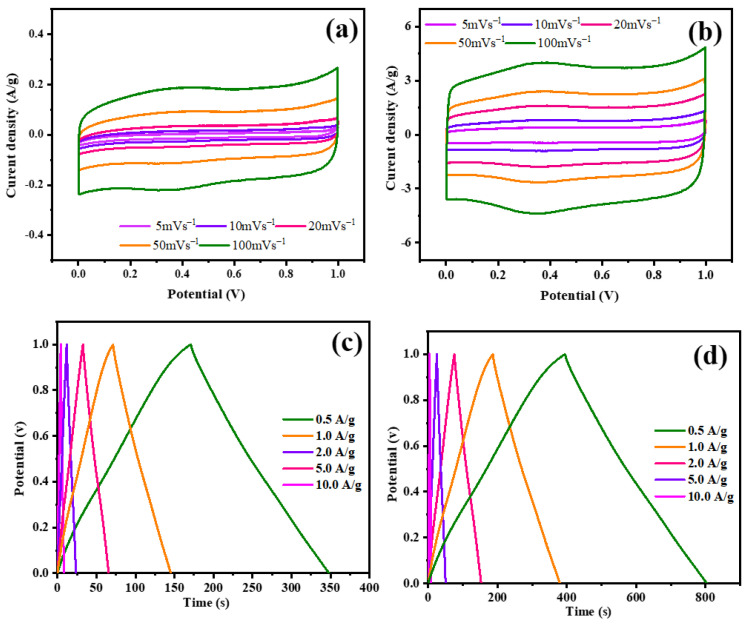
(**a**,**b**) CV curves and (**c**,**d**) GCD curves of PVA/poly (AAm) and PVA/poly (AAm)/PBzC electrodes.

The potential use of PVA/poly (AAm) and PVA/poly (AAm)/PBzC materials for the development of electrode materials was investigated. To assess their suitability, the fabricated hydrogel materials were subjected to electrochemical characterization using a three-electrode system, where a 1 M H_2_SO_4_ aqueous electrolyte served as the testing medium. The CV curves obtained from both the electrodes, i.e., PVA/poly (AAm) and PVA/poly (AAm)/PBzC, at different scan rates are displayed in Figure 7a,b. Both measurements were conducted in the 1 M H_2_SO_4_ aqueous electrolyte with a working voltage window ranging from 0.0 to 1.0 V. Intriguingly, the CV curves for both the electrodes exhibited a quasi-rectangular profile within the voltage range of 0.0−1.0 V and at various scan rates spanning from 5 to 100 mV s^−1^. Notably, these curves retained almost a perfect rectangular shape at the slowest scan rate of 20 mV s^−1^. These observations strongly suggest that the prepared materials possess excellent capacitive behavior [32,33,34,35,36,37,38]. Further analysis revealed a dependence of the curve shapes on the scan rate. At a higher scan rate of 100 mV s^−1^, a slight deviation from the rectangular shape was observed in PVA/poly (AAm)/PBzC (Figure 7b), which confirms the occurrence of pseudo-capacitive reactions, potentially attributable to the higher nitrogen content in the materials. Interestingly, even at this higher scan rate, both the electrodes maintained a form that deviated only slightly from a rectangle [39,40,41,42]. This continued capacitance behavior implies good performance during charge and discharge operations at faster rates.

Figure 7c,d display the GCD curves of PVA/poly (AAm) and PVA/poly (AAm)/PBzC electrodes, exhibiting a linear shape with a slight curvature. This indicates good capacitive properties and electrochemical reversibility of the electrodes, where the energy can be stored and released effectively and in a predictable manner. Among these electrodes, the PVA/poly (AAm)/PBzC electrode stands out with a significantly longer discharge time compared to PVA/poly (AAm). This extended discharge time can be attributed to the presence of macro-pores and meso-pores within the PBzC component. This kind of interconnected porous morphology offers two key benefits. Firstly, the pores act like highways, allowing electrolyte ions to reach most of the electrode’s inner surface area. This larger accessible area translates to more sites for ions to be stored, thereby increasing the electrode’s capacitance. And secondly, these pore channels facilitate the rapid movement of electrolyte ions throughout the electrode, even at high current densities. This efficient movement ensures the electrode can deliver its stored charge quickly when needed. In simple terms, the macro-pores and meso-pores in the PVA/poly (AAm)/PBzC electrode create a highly porous structure with a high concentration of electrochemically active nitrogen and oxygen atoms. The activated porous carbons contain higher contents of pyridinic and pyrrolic nitrogen species, possibly obtained from the polybenzoxazine source. These nitrogen species become electrochemically active in an acidic aqueous solution, thereby increasing capacitance. Graphitic nitrogen species present in the interior of the carbon matrix effectively promote electron transfer, thus improving conductivity. These nitrogen and oxygen atoms contribute to pseudo-capacitance, thereby significantly boosting the electrode’s overall capacitance [43,44,45,46].

**Figure 8:**
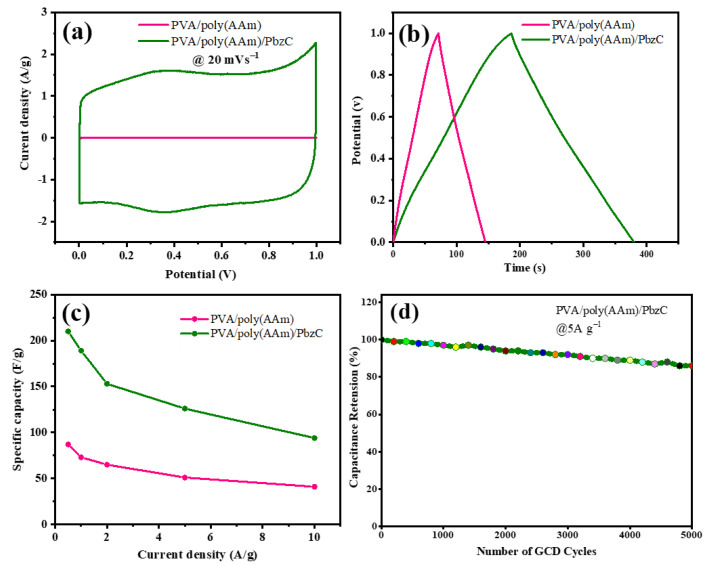
(**a**) CV, (**b**) GCD, (**c**) specific capacitance, and (**d**) cyclic stability of the electrodes.

The findings from both GCD and CV measurements are consistent with each other, supporting the conclusions drawn above. Figure 8a,b further reinforce this point. The CV curves at a scan rate of 20 mV s^−1^ and the GCD curves at a current density of 1 A g^−1^ of PVA/poly (AAm) and PVA/poly (AAm)/PBzC electrodes were compared. The curve clearly demonstrates that the PVA/poly (AAm)/PBzC electrode delivers a substantially larger CV area and longer discharge time due to its wider distribution of micro-pores and the presence of additional meso- and macro-pores [47,48,49]. All these results strongly suggest that the presence of PBzC in the electrode significantly enhances its pseudo-capacitance, leading to superior performance compared to the PVA/poly (AAm) electrode. Figure 8c illustrates the significant improvement in specific capacitance achieved by incorporating PBzC into the PVA/poly (AAm) electrode. At a current density of 0.5 A g^−1^, a specific capacitance (C_s_) of 92 F g^−1^ was obtained for the PVA/poly (AAm) electrode. A remarkable improvement was obtained after the incorporation of PBzC, with a Cs of 210 F g^−1^ for the PVA/poly (AAm)/PBzC electrode. This substantial enhancement can be attributed to the porous structure of the PBzC material, which facilitates efficient electrolyte penetration and maximizes the electrode surface area accessible for ion interaction. This effectively addresses a key requirement for high capacitance and positions the PVA/poly (AAm)/PBzC electrode as a promising candidate for supercapacitor applications.

To further investigate the potential of PVA/poly (AAm)/PBzC electrodes, their cycling stability was explored through galvanostatic charge/discharge (GCD) study and is depicted in Figure 8d. The experiment employed a three-electrode system in a 1 M H_2_SO_4_ aqueous electrolyte at a current density of 5 A g^−1^. Notably, the C_s_ of the PVA/poly (AAm)/PBzC electrode exhibited excellent stability over 5000 charge/discharge cycles. Although a slight decrease from 126 to 110 F g^−1^ was observed at a current density of 5 to 10 A g^−1^ (Figure 8c), the capacitance retention ratio remained impressive at 87% (Figure 8d). This remarkable stability highlights the exceptional long-term durability of PVA/poly (AAm)/PBzC electrodes, making them highly suitable for practical supercapacitor devices.

**Figure 9:**
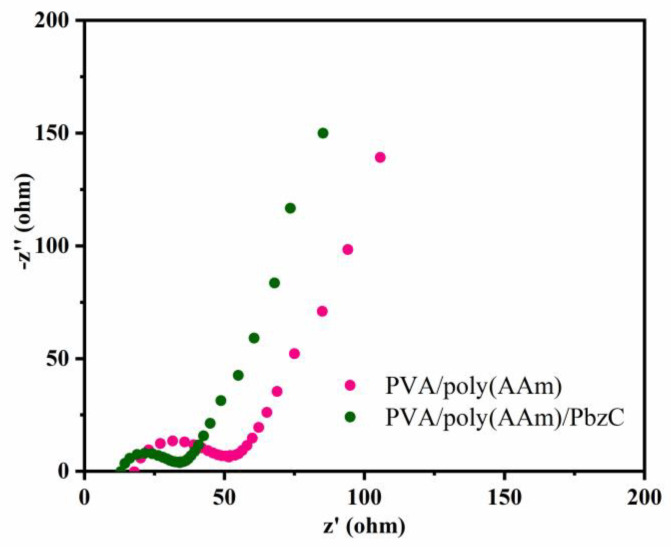
Impedance measurement of the electrodes.

Figure 9 illustrates Nyquist plots for both the electrodes under study. Notably, the plots for both electrodes exhibited a similar pattern. At high frequencies, a depressed semicircle was observed, indicating the presence of a capacitive behavior. Conversely, a gradual slope emerged at low frequencies, further affirming this capacitive behavior. Compared to the PVA/poly (AAm) samples, the Nyquist plot for the PVA/poly (AAm)/PBzC electrode displayed a shorter semicircle with a less steep slope, implying lower resistance to diffusion. This observation suggests enhanced ionic conductivity due to the presence of meso- and macro-pores, with a high degree of graphitization in the PVA/poly (AAm)/PBzC electrode [50,51]. Moreover, the charge transfer resistances for the PVA/poly (AAm) and PVA/poly (AAm)/PBzC electrodes were found to be 35.9 and 22 Ω, respectively. These results further support the superior ionic conductivity of the PVA/poly (AAm)/PBzC electrode compared to the other electrodes (Table 1).

## 4. Conclusions

Polybenzoxazine, a novel class of high-performance thermosetting resin, was used as a carbon source for producing a hetero-atom-derived carbon material. The carbon material produced has a suitable pore size with interconnected channels of micro-, meso-, and macro-pores. The dual property of the carbon material with hetero-atom doping and a porous morphology gains importance when used as an electrode material in supercapacitors. The flexibility aspect in supercapacitor is brought about by producing a composite polymer hydrogel consisting of PVA, polyacrylamide, and PBzC. The presence of a hetero-atom and interconnected pore channels gives rise to additional capacitance resulting from both the EDLCs and pseudo-capacitance behavior. The composite polymer hydrogel, PVA/poly (AAm)/PBzC, demonstrates high stiffness with a tensile strength of 7.8 kPa and a compressive strength of 39 kPa. Additionally, the specific capacitance is maintained even at a higher current density [Cs of PVA/poly (AAm)/PBzC = 210 F g^−1^ @ 0.5 A g^−1^ and 110 F g^−1^ @ 10 A g^−1^] along with a capacitance retention of 87%, even after 5000 cycles. Overall, these electrochemical studies demonstrate the promising potential of PVA/poly (AAm)/PBzC as a flexible electrode for supercapacitor applications due to its excellent capacitive behavior and good rate capability.

## Figures and Tables

**Table 1:** Summary of the electrochemical performance of other graphene-based hydrogels as electrode materials.

Electrode Material	Electrolyte	Specific Capacitance	References
N-doped graphene hydrogel	PVA/H_2_SO_4_	61.7 F/g	[52]
3D graphene hydrogel	PVA/H_2_SO_4_	186.3 F/g	[53]
N,F-doped graphene hydrogel	PVA/KOH	170.2 F/g	[2]
GO/PNIPAM hydrogel	PVA/H_2_SO_4_	292.0 F/g	[54]
rGO/carbon hydrogel	PVA/H_2_SO_4_	264 F/g	[55]
N-doped graphene/carbon hydrogel	PVA/KOH	197 F/g	[56]
PVA/poly (AAm)/PBzC	H_2_SO_4_	210 F/g	This work

## Data Availability

The original contributions presented in this study are included in the article, further inquiries can be directed to the corresponding authors.
